# Identification and Validation of a Prognostic Model Based on Three MVI-Related Genes in Hepatocellular Carcinoma

**DOI:** 10.7150/ijbs.66536

**Published:** 2022-01-01

**Authors:** Yongchang Tang, Lei Xu, Yupeng Ren, Yuxuan Li, Feng Yuan, Mingbo Cao, Yong Zhang, Meihai Deng, Zhicheng Yao

**Affiliations:** 1 Department of Hepatobiliary Surgery, The Third Affiliated Hospital, Sun Yat-sen University, Guangzhou, 510630, China.; 2 Department of Nuclear Medicine, The Seventh Affiliated Hospital, Sun Yat-sen University, Shenzhen, 518107, China.; 3 Department of Nuclear Medicine, The Third Affiliated Hospital, Sun Yat-sen University, Guangzhou, 510630, China.; 4 Department of General Surgery, The Third Affiliated Hospital, Sun Yat-sen University, Guangzhou, 510630, China.

**Keywords:** Hepatocellular Carcinoma, MVI, Prognostic Model, MVI-related Gene

## Abstract

MVI has significant clinical value for treatment selection and prognosis evaluation in hepatocellular carcinoma (HCC). We aimed to construct a model based on MVI-Related Genes (MVIRGs) for risk assessment and prognosis prediction in patients with HCC. This study utilized various statistical analysis methods for prognostic model construction and validation in the Cancer Genome Atlas (TCGA) and International Cancer Genome Consortium (ICGC) cohorts, respectively. In addition, immunohistochemistry and qRT-PCR were used to analyze and identify the value of the model in our cohort. After the analyses, 153 differentially expressed MVIRGs were identified, and three key genes were selected to construct a prognostic model. The high-risk group showed significantly lower overall survival (OS), and this trend was observed in all subgroups: different age groups, genders, stages, and grades. Risk score was a risk factor independent of age, gender, stage, and grade. Moreover, the ICGC cohort validated the prognostic value of the model corresponding to the TCGA. In our cohort, qRT-PCR and immunohistochemistry showed that all three genes had higher expression levels in HCC samples than in normal controls. High expression levels of genes and high-risk scores showed significantly lower recurrence-free survival (RFS) and OS, especially in MVI-positive HCC samples. Therefore, the prognostic model constructed by three MVIRGs can reliably predict the RFS and OS of patients with HCC and is valuable for guiding clinical treatment selection and prognostic assessment of HCC.

## Introduction

HCC is one of the most common malignancies of the digestive system worldwide. It has an insidious onset, easy recurrence and metastasis, and often has a poor prognosis due to the lack of effective prediction and treatment strategies. Thus, it is listed as the third leading cause of cancer death 1-4. A variety of methods have been adopted to prevent and treat HCC, such as the use of hepatitis vaccines, application of targeted immune drugs, neoadjuvant therapy, surgical resection, and liver transplantation 5-9. These methods and the application of some biomarkers have improved the therapeutic effect of HCC to a certain extent, but their prognosis remains poor 10. Even after curative treatment, many patients with HCC still experience tumor recurrence within 5 years 11. Although the incidence of the disease has decreased, the specific mortality rate of the disease remains high 12. Therefore, there is an urgent need to develop new prognostic evaluation methods to predict the clinical prognosis of patients with HCC. Constructing a prognostic model for predicting survival and stratifying patient predictions is still of great significance.

Microvascular invasion (MVI) refers to tumor cell clusters which are seen in the vascular cavity lined by endothelial cells under a microscope 13. Tumor cells can exist in the hepatic portal vein or hepatic vein as a potential factor for intrahepatic or distant metastasis, and can prompt postoperative recurrence 14. Studies have shown that MVI is an independent histopathologic prognostic factor related to the survival of patients with HCC at all stages 15. Moreover, MVI is a key factor and an important indicator for predicting early recurrence and survival after HCC surgery 16, 17. Accurate prediction of MVI before surgery can help clinicians make more reasonable treatment decisions to truly achieve individualized treatment based on tumor biological behavior. In recent years, although the clinical value of MVI in HCC treatment selection and prognosis assessment has received increasing attention 18-22, since the diagnosis of MVI is mainly based on postoperative pathological examination and there are still no recommended MVI-related molecular markers to predict the prognosis of HCC, it limits the application of MVI to guide the diagnosis and treatment of liver cancer.

In this study, we explored new MVI-related biomarkers and established a risk scoring model for predicting the prognosis of HCC, with the aim of providing suitable treatments for patients with HCC.

## Materials and Methods

### Tissue samples

This study was approved by the Clinical Research Ethics Committee of the Third Affiliated Hospital of Sun Yat-sen University, and informed consent was obtained from all participants. This study used HCC tissues and matched paracancerous tissue samples that were surgically resected at the Third Affiliated Hospital of Sun Yat-sen University between December 2012 and September 2018. The follow-up date was until July 2021. All postoperative pathology reports of tissue samples confirmed the presence of HCC. The samples were then suspended in liquid nitrogen. A part of the tissue sample was fixed in 10% formalin solution and then embedded in paraffin for long-term preservation. They were made into 4 μm thick tissue sections for immunohistochemical staining.

### Data Acquisition and Preprocessing

Two sets of HCC RNA-seq data were collected for this study. Genome-wide mRNA expression and clinicopathological information of HCC were downloaded as training cohort from UCSC Xena the Cancer Genome Atlas liver hepatocellular carcinoma cohort (TCGA LIHC). A total of 95 cancer tissues with MVI, 210 cancer tissues without MVI, and 58 normal tissues, which matched with clinical data, were collected. Liver Cancer - RIKEN, JP Project from International Cancer Genome Consortium (ICGC LIRI-JP) transcriptomic expression data were downloaded as a validation cohort. A total of 232 cancer tissues with follow-up data were collected. For normalization, gene expression quantified with fragment per kilobase million (FPKM) was transformed into transcripts per million (TPM) values and processed by log2(value+1) for all samples before further analysis.

### Identification of differential expressed MVI-related genes in TCGA cohort

Cancer tissues with or without MVI were used for the difference analysis with normal tissues, respectively. The Wilcox test was used to identify differentially expressed genes (DEGs) according to the criteria of |log 2 (fold change) |> 1 and false discovery rate (FDR) < 0.01. We took the difference genes that are in DEGs (cancer tissues with MVI vs. normal tissues) but not in DEGs (cancer tissues without MVI vs. normal tissues) as MVI-related differentially expressed genes.

### Go and KEGG analysis

To further understand the potential biological mechanisms of, we applied 153 MVI-related genes to Gene ontology (GO) and Kyoto Encyclopedia of Genes and Genomes (KEGG) pathway enrichment analyses by WebGestalt (http://www.webgestalt.org/).

### Prognostic signature development and evaluation for HCC

A prognostic signature was constructed based on the training set, followed by validation of its predictive performance in the validation set. Univariate Cox proportional hazard regression analysis was first conducted to evaluate the correlation between MVI-related genes and OS in the training set. With a cutoff value of *p* < 0.01, prognosis-related genes were identified. The Least Absolute Shrinkage and Selection Operator (LASSO) penalized Cox proportional hazards regression (with R packages “glmnet”) were utilized to reduce the genes of the model and limit the complexity of solving the problem of overfitting. Stepwise Cox regression analysis based on the Akaike information criterion (AIC) was used to identify the optimal genes that were used to construct the risk model to predict the prognosis of patients with HCC. The risk score of each patient with HCC was calculated using the following formula: risk score = expression level of gene a * coefficient a + expression level of gene b * coefficient b + expression level of gene c * coefficient c + …… + expression level of gene n * coefficient n.

To evaluate the predictive performance of the model, setting the median risk score as the cutoff value, the patients were classified into a high-risk group and a low-risk group. Kaplan-Meier survival curves were applied for survival comparison between low- and high-risk groups, and log-rank *P* value < 0.05 was regarded as statistically significant (with R packages “survival” and “survminer”). Additionally, time-dependent receiver operating characteristic curves (including 1-, 3-, and 5-year survival) were established (with R package “survival ROC”) to reflect the sensitivity and specificity of the signature.

### Clinical characteristics of the signature

To determine the association of risk scores with clinical features (age, gender, stage, grade, and differentiated grade), we applied the Wilcoxon test for evaluation. The prognostic value of each MVIRG was determined using the same method to calculate the risk score for each case. In addition, we applied the signature to subgroups of patients with different clinical characteristics using the same method to distinguish low- and high-risk cases. Kaplan-Meier survival curves and log-rank tests were also performed.

### Identification of the independent prognostic role of the constructed signature

To further explore the independent prognostic role of the prognostic gene signature, multivariate analysis using the Cox regression model method (with R package “survival”) was performed. We incorporated other clinical factors such as gender, age, cancer grade, AFP level, albumin level, and cancer stage. Statistical significance was set at *p* < 0.05. Moreover, sub-analysis for survival in patients with different clinical characteristics (including younger age, older age, male, female, early stage, and advanced stage) was conducted to further assess the prognostic performance of the signature. To facilitate the evaluation of individual prognostic risk, we further enrolled independent factors to build a nomogram, which was assessed using the C-index and calibration curves.

### External validation of the genes in the prognostic gene signature

The ICGC LIRI-JP dataset was used to validate gene signatures. Each patient's risk score was calculated using the above method. A Kaplan-Meier curve was constructed to test the predictive value of the gene signature. Similarly, the independent prognostic role of the gene signature in ICGC LIRI-JP was evaluated using multivariate Cox analysis.

### Immunohistochemistry (IHC)

Tissue paraffin sections were baked at 60 °C for 2 hours, and then placed in xylene for 15 minutes while they were hot for dewaxing. Different concentrations of alcohol (anhydrous alcohol, 95%, 80%, 75%) were used for hydration treatment, and EDTA antigen retrieval solution (pH=8, ZSGB-BIO, China) was used in the pressure cooker for 25 minutes. After cooling in running water, these sections were treated with 3% hydrogen peroxide for 10 minutes to remove endogenous catalase and then soaked with freshly prepared PBS three times for 5 minutes each time. The primary antibody (1:200) was added and incubated overnight at 4 °C in a humidified box. The next day, the primary antibody was washed away with PBS, the secondary antibody was added, and these sections were incubated at 37 °C for 40 minutes. After washing off the secondary antibody with PBS, it was developed with DAB (Dako REAL™), and the nucleus was stained with hematoxylin.

### Quantitative real-time PCR (qRT-PCR)

After tissue grinding, RNA was extracted with RNAiso Plus (Invitrogen, USA) according to the manufacturer's instructions. cDNA was obtained by reverse transcription using a HisScript® III RT SuperMix for qPCR (+gDNA wiper) kit (Vazyme, China). The qPCR experiment was carried out according to the instructions of the ChamQ Universal SYBR qPCR Master Mix kit (Vazyme, China). The operating instrument used was a Roche LightCycler®480. GAPDH was used as an endogenous control, and only one peak of the melting curve of each reaction could be regarded as a valid result. The experiment was repeated three times. Primer sequences used in this study are shown in Supplementary File 1.

### Statistical analysis

In this study, GraphPad Prism 8.0 and R software v4.0.1 were used for the statistical analysis of the experimental data. All experimental data were expressed as mean ± standard deviation. For comparison between the two samples, data with normal distribution and uniform variance were analyzed using Student's t-test; data with uneven variances were analyzed using the Wilcox test. Statistical significance was set at *p* < 0.05.

## Results

### Differentially expressed genes in MVI-positive and MVI-negative HCC tissues

**Figure 1:**
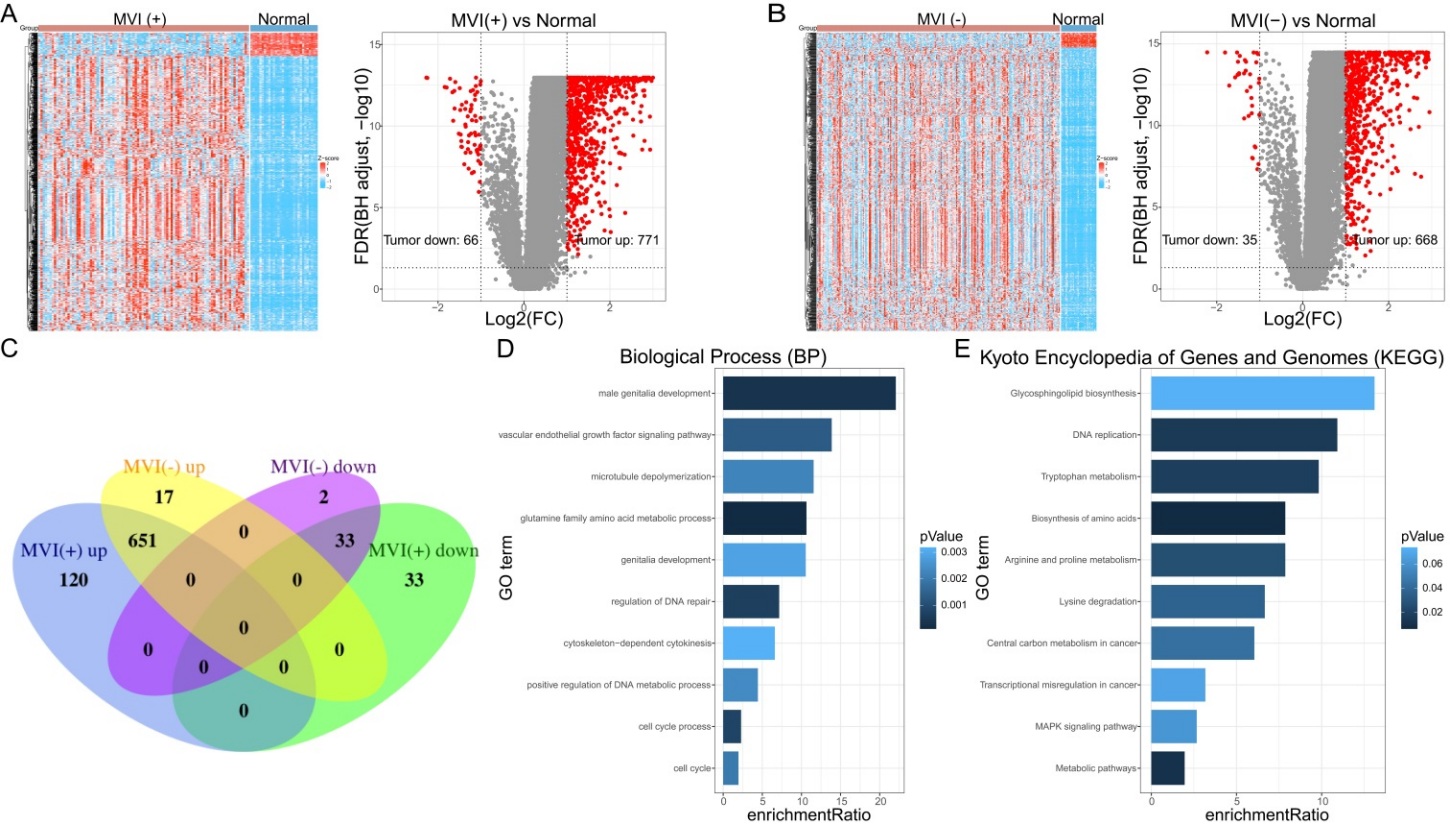
** The DEGs and functional analysis. A:** The hierarchical clustering and the volcano plot of DEGs between HCC tissues with MVI (MVI(+)) and normal tissues. **B:** The hierarchical clustering and the volcano plot of DEGs between HCC tissues without MVI (MVI(-)) and normal tissues. **C:** The venn diagram of 153 identified MVIRGs. **D:** Gene Ontology (GO) enrichment analysis. **E:** Kyoto Encyclopedia of Genes and Genomes (KEGG) analysis. The length of each column represents the count of genes; the shade of color represents the *p* value.

A total of 93 cancer tissue samples with MVI data, 206 cancer tissue samples without MVI data, and 30 normal tissue samples, all of which matched with clinical data, were identified in the TCGA database. Genes with low-expressed (median value <1) were excluded (Supplementary File 2). MVI-positive cancer tissue (MVI (+)) and MVI-negative cancer tissue (MVI (-)) were separately compared with non-cancerous tissue samples to obtain their differentially expressed genes. The results of the difference analysis showed that HCC samples can be clearly distinguished from normal tissues (Figure 1A left, Figure 1B left). The volcano map showed that there are 66 down-regulated DEGs and 771 up-regulated DEGs in MVI-positive cancer tissues (Figure 1A right, Supplementary File 3), and 35 down-regulated DEGs and 668 up-regulated DEGs in MVI-negative cancer tissues (Figure 1B right, Supplementary File 4). Compared with non-cancerous tissues, we recorded the up-regulated DEGs in HCC tissues with MVI as the 'MVI (+) up' group and recorded the up-regulated MVIRGs in HCC tissues without MVI as the 'MVI (-) up' group. Upon comparing the 'MVI (+) up' group and 'MVI (-) up' group, we obtained 120 DEGs. Using the same method, compared with non-cancerous tissues, we recorded the down-regulated DEGs in HCC tissues with MVI as the 'MVI (+) down' group, and recorded the down-regulated DEGs in HCC tissues without MVI as the 'MVI (-) down' group. Upon comparing the 'MVI (+) down' group and 'MVI (-) down' group, we obtained 33 DEGs. These 153 (120+33) DEGs were identified as differentially expressed MVIRGs for subsequent modeling (Figure 1C, Supplementary File 5).

### Functional analysis of differentially expressed MVIRGs

To further study the biological functions of differentially expressed MVIRGs, we performed GO enrichment analysis and KEGG analysis on the 153 MVIRGs. In terms of biological processes (BP), the MVIRGs were mainly involved in the growth of the vascular endothelium and microtubule aggregation (Figure 1D). In terms of the pathways of action, these genes were mainly involved in metabolism-related pathways, tumor transcription disorders, and DNA replication pathways (Figure 1E).

### Construction and identification of prognostic models

**Figure 2:**
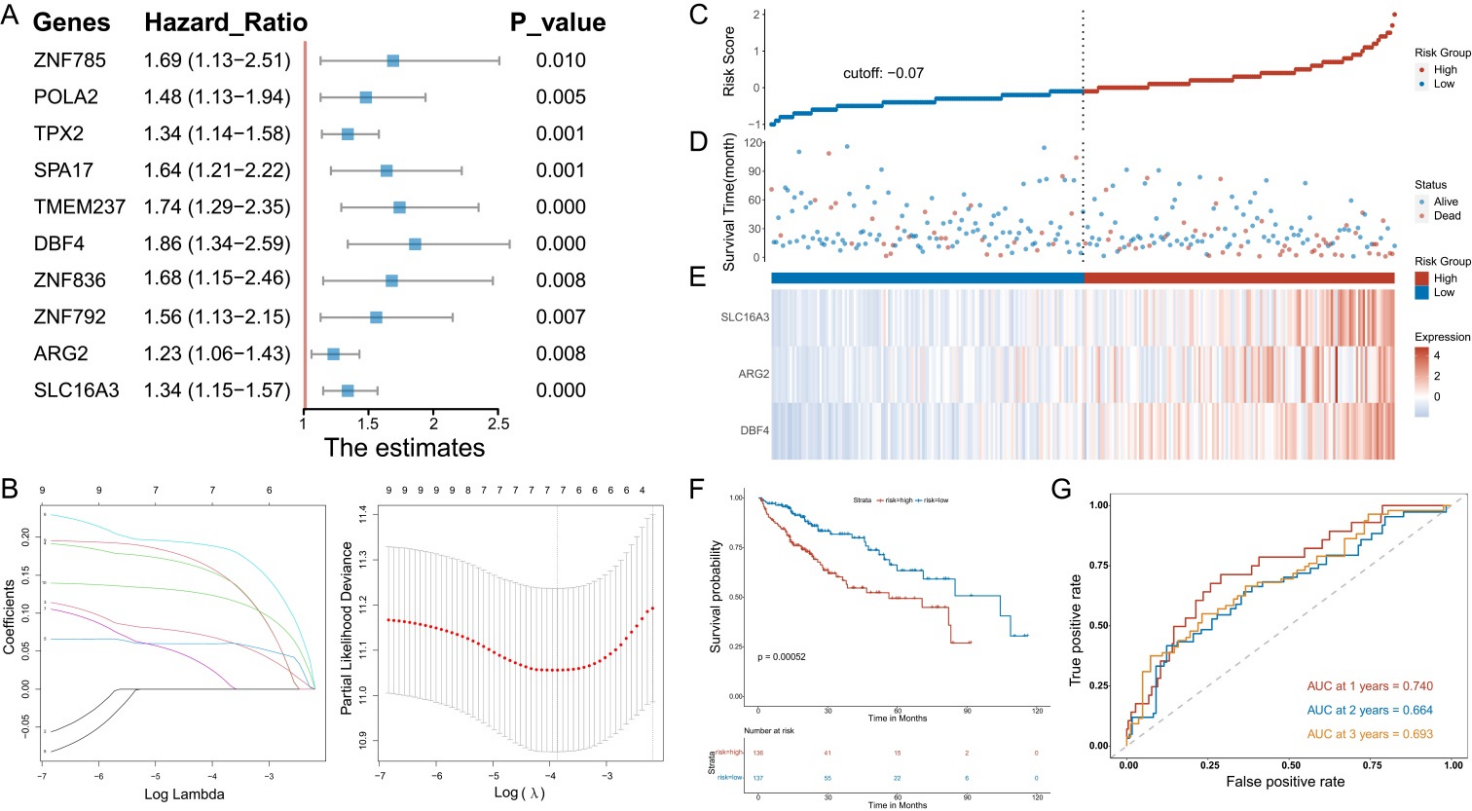
** Stepwise identification of key MVIRGs of the model and prognostic analysis in TCGA cohort. A:** 10 MVIRGs significantly associated with the overall survival (OS) of patients with HCC.** B:** The result of LASSO regression for 10 MVIRGs.** C:** The risk score of each patient with HCC. **D:** The patient survival based on the risk score. **E:** The heat map of the three MVIRGs in the high-risk group and the low-risk group. **F:** Kaplan-Meier plot of patients in a low- or high-risk group (*P* = 0.00052), and the number of patients in different risk groups. **G:** Receiver operating characteristic (ROC) curve analysis for the prognostic value of the prognostic model for different years. Data from TCGA (median risk score as the cut-off value). AUC: area under the curve.

Univariate Cox regression analysis was used to determine the MVIRGs associated with the patients' clinical outcomes. A total of 10 MVIRGs were screened to be significantly associated with the OS of patients with HCC (Figure 2A, Supplementary File 6). Based on the 10 genes associated with prognosis, LOSSO regression and stepwise Cox regression analysis were performed to construct a prognostic model based on MVI. Finally, three key MVIRGs (DBF4, ARG2, and SLC16A3) were selected to construct the model (Figure 2B, Table 1, Table 2). The risk score formula of the model was as follows: risk score = (0.400506 × DBF4 expression) + (0.188240 × ARG2 expression) + (0.204192 × SLC16A3 expression).

The results showed the risk score of each patient with HCC in the TCGA database (Figure 2C), patient survival based on the risk score (Figure 2D), and the heat map of the three MVIRGs in the high-risk group and the low-risk group (Figure 2E). These indicated that as the risk score increased, the expression of three key MVIRGs increased, survival time decreased, and mortality increased. The Kaplan-Meier curve showed that the survival rate of the high-risk group (n = 136) was significantly lower than that of the low-risk group (n = 137) (Figure 2F). Additionally, the ROC curve showed that the AUC values were 0.740 (1 year), 0.664 (2 years), and 0.693 (3 years), indicating that the model performed well in predicting the survival rate of patients with HCC (Figure 2G).

**Figure 3:**
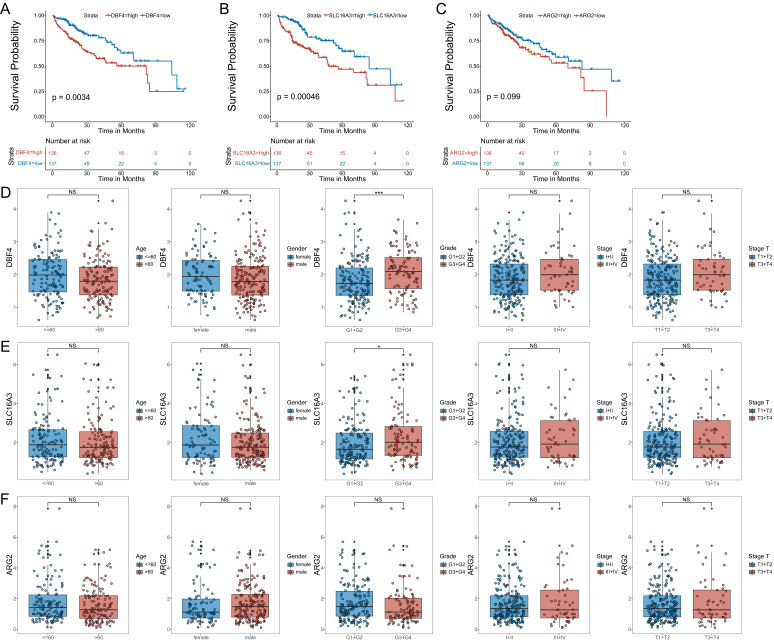
** The correlation between the respective expression levels of the three MVIRGs and the OS and the clinical data in patients with HCC. A:** The Kaplan-Meier plot between the expression level of DBF4 and OS in patients with HCC (*P* = 0.0034), and the number of patients in different groups. **B:** The Kaplan-Meier plot between the expression level of SLC16A3 and OS in patients with HCC (*P* = 0.00046), and the number of patients in different groups. **C:** The Kaplan-Meier plot between the expression level of ARG2 and OS in patients with HCC (*P* = 0.099), and the number of patients in different groups.Date from TCGA (median risk score as the cut-off value). **D:** The correlation between the expression level of DBF4 and the clinical data. **E:** The correlation between the expression level of SLC16A3 and the clinical data. **F:** The correlation between the expression level of ARG2 and the clinical data. Data from TCGA. Clinical data: age (≤60 vs. >60), gender (female vs. male), grade (G1+G2 vs. G3+G4), stage (I+II vs. III+IV), stage T (T1 +T2 vs. T3+T4). NS: not significant; *P < 0.05, **P < 0.01, ***P < 0.001.

**Figure 4:**
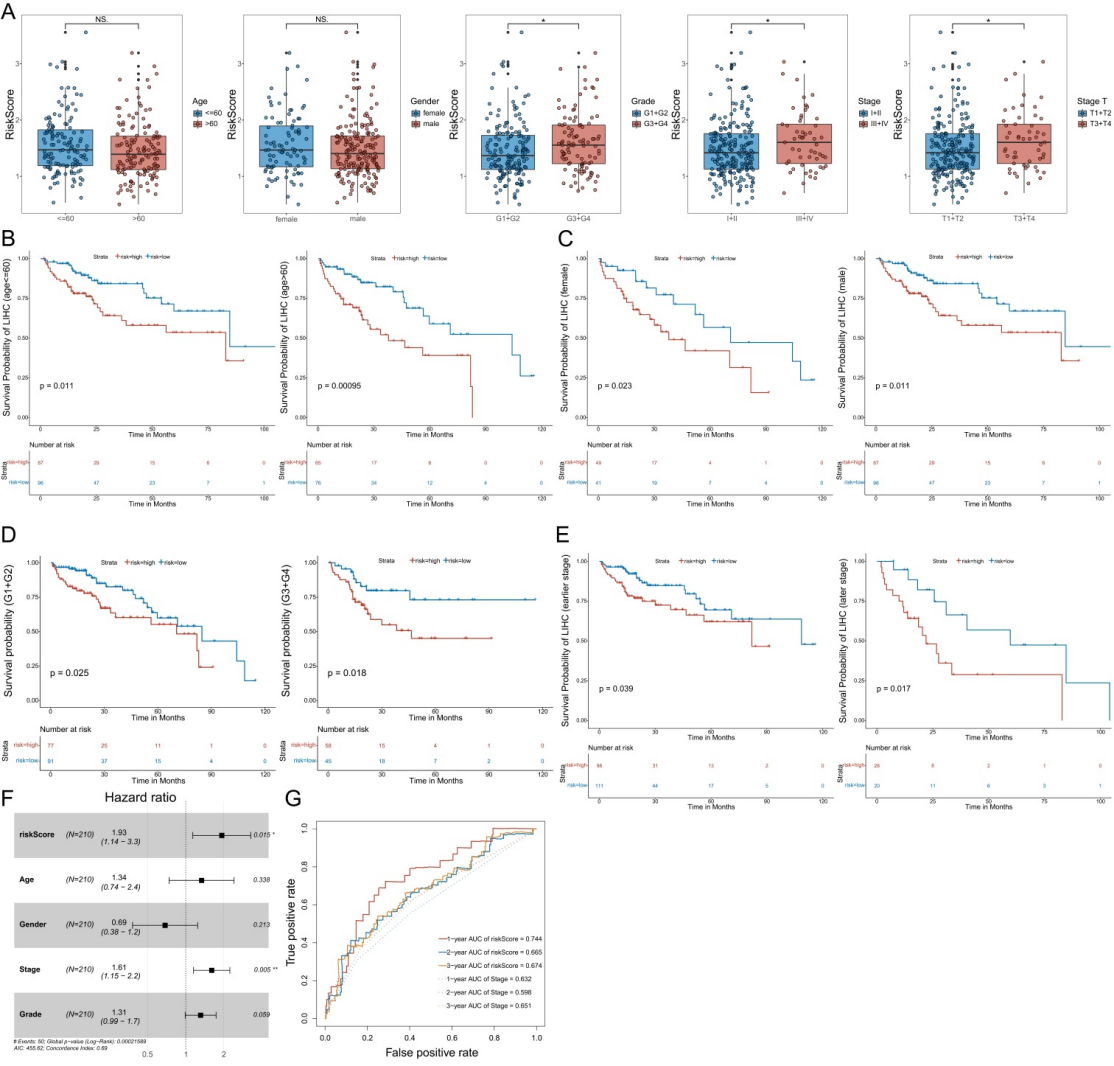
** The correlation between the risk score and the clinical data, and the subgroup analysis, Multivariate Cox regression analyses and ROC curve of OS of the clinical data. A:** The correlation between the risk score and the clinical data. **B:** The Kaplan-Meier analysis of different age groups (≤60 and >60). **C:** The Kaplan-Meier analysis of different gender groups (female and male). **D:** The Kaplan-Meier analysis of different grade groups (G1+G2 and G3+G4). **E:** The Kaplan-Meier analysis of different stage groups (I+II and III+IV). **F:** Multivariate Cox regression analysis. The risk score and stage were independent risk factors for OS. **G:** The ROC curve of the independent risk factors (the risk score and stage) for OS in HCC. Data from TCGA (median risk score as the cut-off value). Clinical data: age (≤60 vs. >60), gender (female vs. male), grade (G1+G2 vs. G3+G4), stage (I+II vs. III+IV), stage T (T1 +T2 vs. T3+T4). Earlier stage: stage I+II, Later stage: stage III+IV. Risk Score (high-risk score vs. low-risk score). NS: not significant; *P < 0.05, **P < 0.01, ***P < 0.001.

To further validate the model, we analyzed the correlation between the expression of the three MVIRGs and clinical parameters in patients with HCC. First, we analyzed the correlation between the respective expression levels of the three MVIRGs and the survival rate of patients with HCC. The Kaplan-Meier curves showed that the high expression of DBF4 and SLC16A3 genes was significantly associated with low OS in patients with HCC (Figure 3A-3B), and high expression of ARG2 may indicate a poor prognosis in patients with HCC, but this was not statistically significant (Figure 3C). Second, we analyzed the correlation between the respective expression levels of the three MVIRGs and clinical data. The results of the Wilcox test revealed that there was a significant correlation between the high expression of DBF4 (*p*<0.001), high expression of SLC16A3 (*p*<0.05), and tumor grade in HCC (Figure 3D-3F). Similarly, we analyzed the correlation between the risk score calculated by the model and clinical data. The analysis showed that the high-risk score was significantly related to cancer grade, stage, and T stage (Figure 4A). At the same time, we conducted a subgroup analysis of the clinical data of the high-risk and low-risk groups to verify the predictive ability of the prognostic model for patients with different clinical characteristics, which showed that there were significant differences in the prognosis according to different age groups (≤60 and >60) (Figure 4B), different gender groups (female and male) (Figure 4C), different grade groups (G1+G2 and G3+G4) (Figure 4D), different stage groups (I+II and III+IV) (Figure 4E). This meant that the prognostic model had good predictive and discriminative abilities.

In addition, we determined whether the risk score or conventional clinical parameters of patients with HCC were independent risk factors for OS. The results of multivariate Cox regression analysis showed that the risk score and stage were independent risk factors for OS (Figure 4F). We used the ROC curve to compare the predicted value of the risk score with other clinical parameters. The results showed that stage had the highest predictive value among the conventional clinical parameters. However, the predicted value of the risk score was better than that of the stage (Figure 4G).

### Confirmation of the prognostic model using an additional data cohort (ICGC)

**Figure 5:**
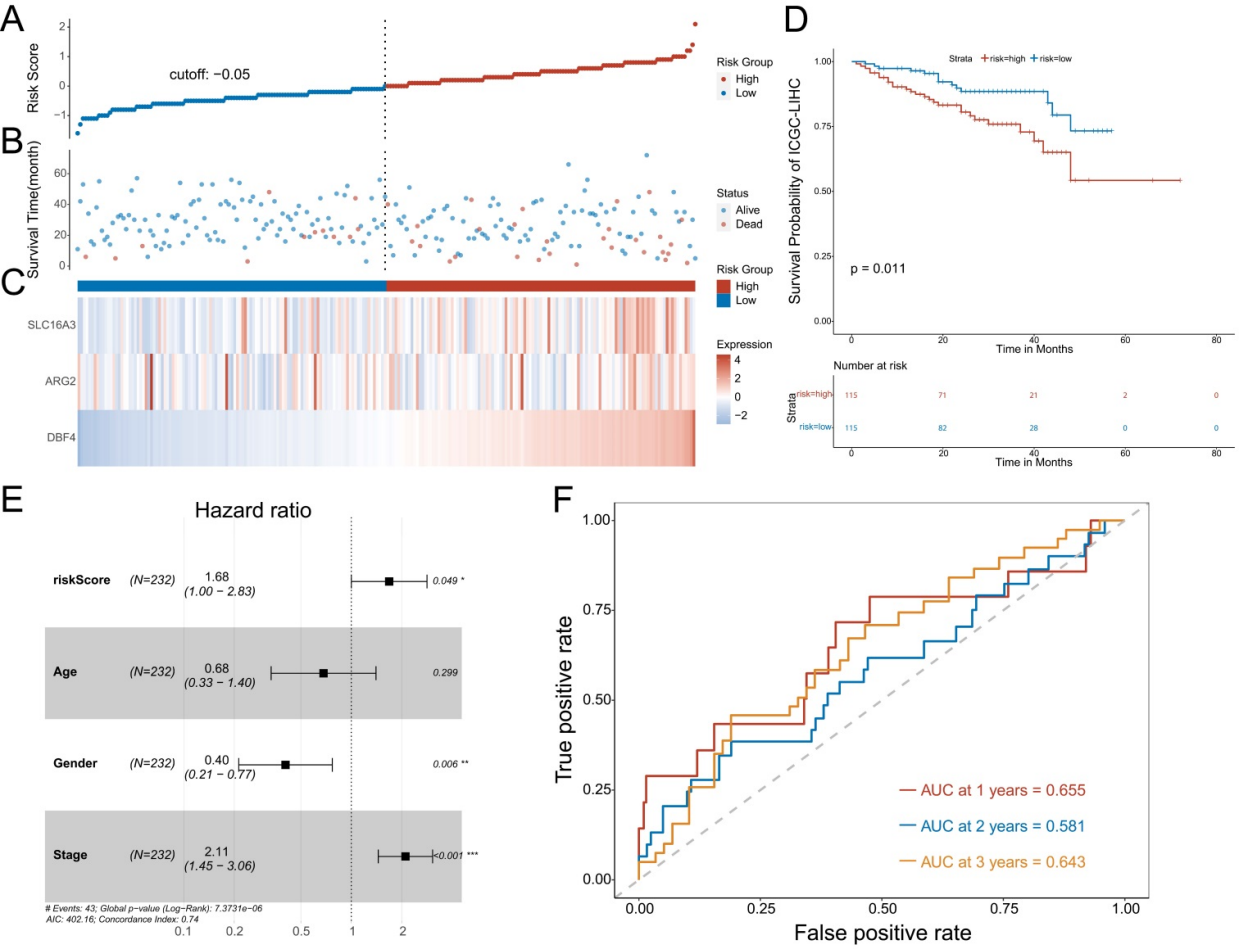
**Further validation of the model in ICGC cohort. A:** The risk score of each patient with HCC. **B:** The patient survival based on the risk score. **C:** The heat map of the three MVIRGs in the high-risk group and the low-risk group. **D:** The Kaplan-Meier plot of patients in a low- or high-risk group (*P* = 0.011), and the number of patients in different risk groups. **E:** Multivariate Cox regression analysis. The risk score and stage were independent risk factors for OS. **F:** ROC curve analysis for the prognostic value of the prognostic model for different years. Data from ICGC (median risk score as the cut-off value). AUC: area under the curve. *P < 0.05, **P < 0.01, ***P < 0.001.

To further confirm the predictive value of the prognostic model, we adopted the same method to divide patients with HCC into low-risk or high-risk groups in the ICGC cohort. The results showed the risk score of each patient with HCC in the ICGC database (Figure 5A), patient survival based on the risk score (Figure 5B), and the heat map of the three MVIRGs in the high-risk group and the low-risk group (Figure 5C). These results were consistent with the trend of the results in the TCGA cohort. Kaplan-Meier analysis showed a significant difference between the two groups (*p* = 0.011) (Figure 5D), consistent with the trend observed in the TCGA cohort. In addition, the multivariate Cox regression analyses of ICGC data also showed that the risk score and stage were independent risk factors for OS in patients with HCC (Figure 5E). The ROC curve showed that the AUC values were 0.655 (1 year), 0.581 (2 years), and 0.643 (3 years), verifying that the predictive model had good predictive value (Figure 5F).

### Verification of clinical tissue samples

**Figure 6:**
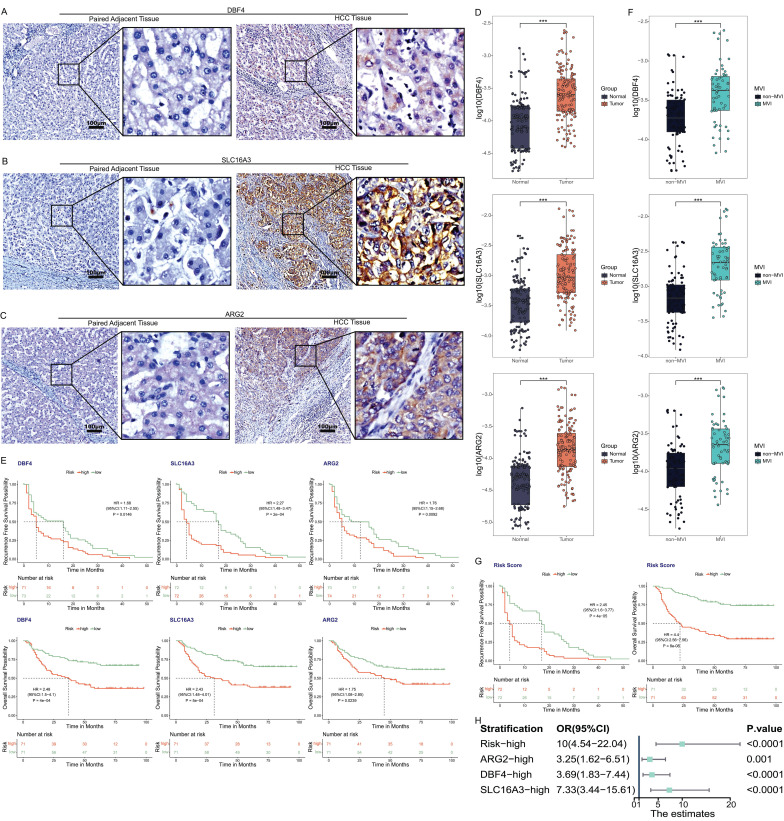
** The expression of three MVIRGs in tissue samples. A:** Immunohistochemical staining of DBF4 in HCC tissues (right) and paired adjacent tissues (left). **B:** Immunohistochemical staining of SLC16A3 in HCC tissues (right) and paired adjacent tissues (left). **C:** Immunohistochemical staining of ARG2 in HCC tissues (right) and paired adjacent tissues (left). **D:** The expression of these three MVIRGs in tissue samples. **E:** The RFS (upper) and OS (under) of these three MVIRGs in tissue samples. **F:** The expression of these three MVIRGs in tissue samples with or without MVI. **G:** The RFS (left) and OS (right) of the risk score in tissue samples. **H:** The forest plot analysis of these three MVIRGs and the risk score. Median value as the cut-off value. *P < 0.05, **P < 0.01, ***P < 0.001.

The immunohistochemistry results of 34 pairs of tissue sections of HCC tissues and paired adjacent tissues showed that the characteristic proteins corresponding to the three MVIRGs (DBF4, ARG2, and SLC16A3) were more strongly stained and more positive areas in cancer tissues than in adjacent tissues (Figure 6A-6C).

The qRT-PCR results of 156 pairs of samples showed that the expression levels of the three MVIRGs (DBF4, ARG2, and SLC16A3) in HCC samples were higher than those in the paired adjacent tissues (Figure 6D). At the same time, the follow-up data were analyzed (excluding the data of some patients who were lost to follow-up) and it was found that the RFS and OS rates of high expression levels were lower than those of low expression levels (Figure 6E). Moreover, we found that the expression levels of these three MVIRGs in MVI-positive (MVI group) HCC samples were higher than those in MVI-negative (non-MVI group) HCC samples (Figure 6F). To verify the correctness of the model in the sample qRT-PCR data, we standardized the risk score formula of the model using the following scaled formula: risk score = (0.269449 × DBF4 expression) + (0.240198 × ARG2 expression) + (0.244787 × SLC16A3 expression). We calculated the risk score of these patients, and used the median risk score as a cut-off value to classify them into high-risk group or low-risk group. The results showed that the RFS and OS rates of the high-risk group were worse (Figure 6G). We further conducted a forest plot analysis of the results and found that the high expression of these three MVIRGs and the higher risk scores were more likely to lead to the occurrence of MVI. The risk score had the greatest impact among them (Figure 6H).

### Construction and identification of nomogram model for individualized evaluation

**Figure 7:**
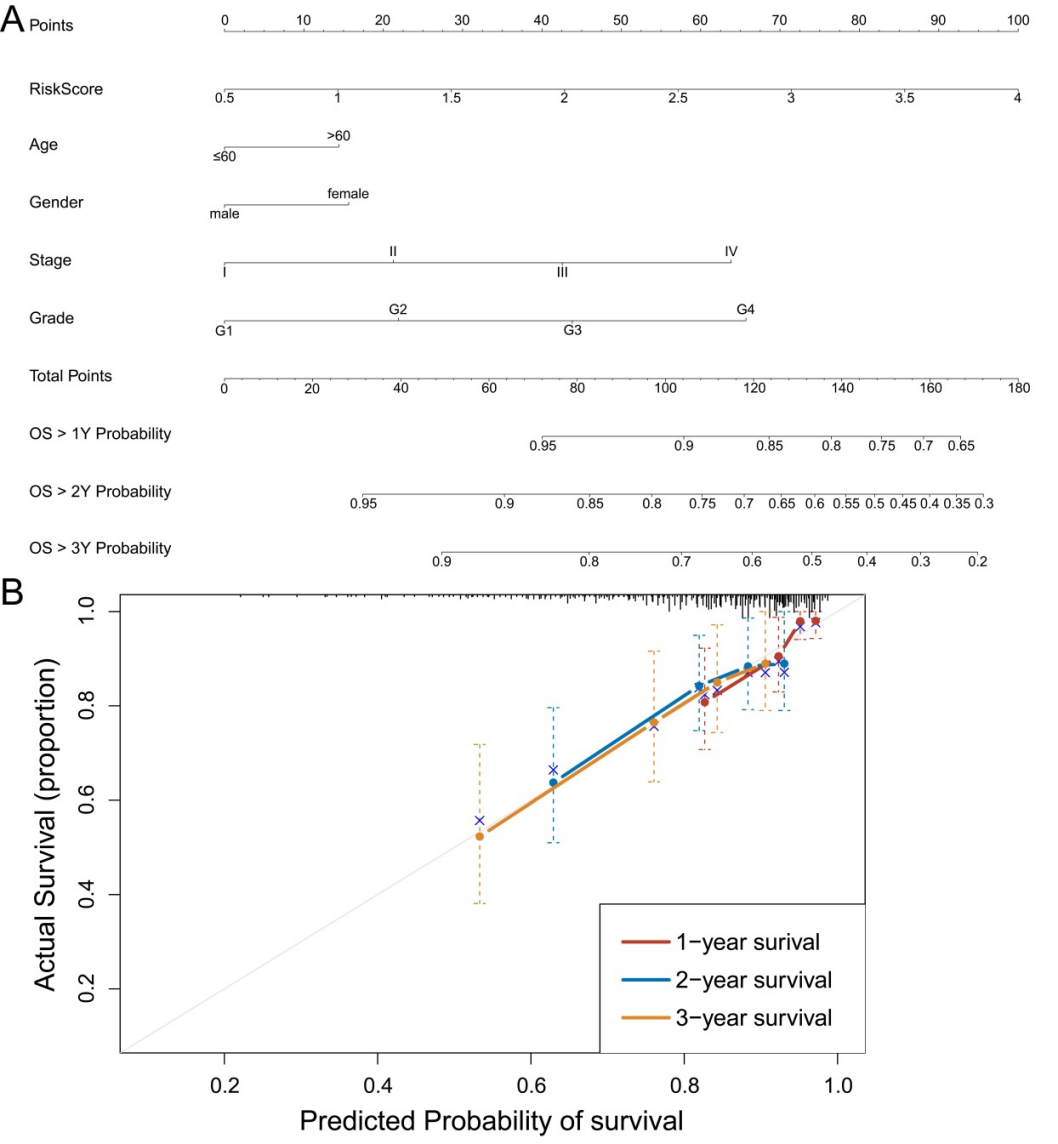
** Construction and identification of nomogram model. A:** Nomogram model, with c-index=0.697, 95% CI (0.632, 0.762). **B:** The calibration curve of the nomogram model. Data from TCGA.

To further make specific predictions of individual prognosis, we introduced conventional clinical parameters into the model and constructed a nomogram model, with a c-index of 0.697 (95% CI (0.632, 0.762)) (Figure 7A). Moreover, the calibration curve verified that the model had a good predictive value (Figure 7B).

## Discussion

HCC is a common malignant tumor of the digestive system and is one of the leading causes of cancer-related deaths worldwide 23. In terms of the pathophysiologic process, HCC has extremely malignant characteristics, which are mainly manifested in the rapid development of cancer, poor conventional treatment effects, poor sensitivity to radiotherapy and chemotherapy, easy metastasis, and high recurrence rate. In addition to the insidious characteristics of this disease, patients with HCC have usually entered the middle and advanced stages of the disease upon diagnosis, thus the prognosis is poor 24, 25. In recent years, the treatment of HCC has substantially improved, and systemic treatment has made great progress; however, the management of this disease is becoming increasingly complicated 23. Despite the decrease in incidence, the specific mortality rate of the disease remains high 26. Therefore, there is an urgent need to develop new evaluation methods to predict the clinical prognosis of patients with HCC.

MVI is an important predictor of survival in patients with HCC 27. In recent years, an increasing number of studies have found that MVI plays an important role in guiding the treatment of HCC 28-33. Some scholars even pointed out that MVI can better predict tumor recurrence and OS than the Milan criteria 34. Although the predictive effect of MVI on the prognosis of HCC is obvious, there are still no recommended MVI-related molecular markers to reliably predict the prognosis of HCC. Based on this, we combined MVI-related genes with conventional clinical parameters to construct a prognostic model that may have a better predictive value for patients with HCC.

In this study, we first used high-throughput sequencing data from TCGA to construct a prognostic model and verified it. By comparing the sequencing data of HCC tissues with or without MVI, we identified 153 differentially expressed MVIRGs. GO and KEGG analyses showed that these MVIRGs were mainly enriched in the growth of vascular endothelium and microtubule accumulation, and mainly affected the prognosis of HCC through metabolic pathways, tumor transcription disorder pathways, and DNA replication pathways. These results were consistent with the results of previous studies. For example, MVI means that tumor cell clusters are seen in the vascular cavity lined by endothelial cells, which indicates that it is related to the growth of vascular endothelium and the accumulation of microtubules 13, 14. Furthermore, MVI is closely related to epithelial-mesenchymal transition (EMT), which involves a variety of mechanisms, including metabolic changes, transcriptional regulation, and epigenetic abnormalities, indicating that MVI is also related to these pathways 35-42.

Univariate Cox regression analysis identified 10 MVIRGs that were significantly associated with the OS of patients with HCC. To prevent overfitting, LASSO regression and stepwise Cox regression analysis were used to select three key MVIRGs (DBF4, ARG2, and SLC16A3) to construct the model. Here, the three genes used for modeling have been shown to be closely related to tumors. Among them, DBF4 was found to be essential for DNA replication in eukaryotes. The protein it encodes has a modular architecture 43, and plays a key role in DNA replication 44, activation of the replication checkpoint 45, meiosis 46, mitotic exit 47, translesion synthesis 48, and histone homeostasis 49. More than 10 years ago, some scholars pointed out that higher ASK/Dbf4-expressing melanomas were associated with lower relapse-free survival 50. Inhibition of the Cdc7/Dbf4 kinase activity can affect specific phosphorylation sites on MCM2 in cancer cells 51. Dorine et al. 52 found that approximately 50% of cell lines had increased Cdc7 protein expression by examining 62 human tumor cell lines. Most of these cell lines had increased Dbf4 abundance, and some had extra copies of the DBF4 gene. Cheng et al. 53 confirmed that Cdc7-Dbf4-mediated phosphorylation of HSP90-S164 can stabilize the HSP90-HCLK2-MRN complex to enhance ATR/ATM signaling that overcomes replication stress in cancer. ARG2 (Arginase 2) is a protein-coding gene, and an increase in its activity is usually associated with more advanced diseases and poor clinical prognosis, in addition to being closely related to tumor immune response 54-56. Recent studies have found that overexpression of ARG2 is a poor prognostic factor in a variety of cancer types, including pancreatic cancer 57, thyroid tumors 58, gastric cancer 59, neuroblastoma 60, 61, head and neck squamous cell carcinoma 62, and acute myeloid leukemia (AML) 63. Tamara et al. 64 found that silencing or the absence of ARG2 can lead to the accumulation of ammonia and inhibition of growth of obesity-associated pancreatic cancer. One study confirmed that mitochondrial ARG2 is a cell‑autonomous regulator of CD8^+^ T cell function and antitumor efficacy 65. However, another study showed that ARG2 has a cancer suppressor effect. It can suppress renal carcinoma progression via the biosynthetic cofactor pyridoxal phosphate depletion and increased polyamine toxicity 66. SLC16A3 (Solute Carrier Family 16 Member 3) is a protein-coding gene. The monocarboxylate transporter 4 (MCT4) encoded by the SLC16A3 gene can catalyze the proton-linked transport of monocarboxylates, which participate in many metabolic processes in the body and can produce anti-apoptotic effects 67, 68. MCT4 is found prominently in glycolytic tissues, such as hypoxic cancer cells, overexpressed in some cancer cells, and plays a critical role in cancer cell growth and proliferation 69-71. Zhang et al. 72 verified that SLC16A3 is an independent indicator of poor prognosis and metastasis in patients with lung adenocarcinoma. Yu et al. 73 also revealed that SLC16A3 is a key regulator of the metabolic process in pancreatic cancer through bioinformatics methods. A comprehensive analysis of DNA methylation data revealed that SLC16A3 has excellent predictive power for tumor diagnosis and prognosis 74. Some studies have also clarified the new epigenetic mechanism of SLC16A3 promoter DNA methylation and/or MCT4 protein levels in thyroid cancer and clear cell renal cell carcinoma, which can provide a biological basis for clinical prognosis 68, 75, 76. Furthermore, many studies have found that the expression level of MCT4 is closely related to the progression of tumors, such as hepatocellular carcinoma 77, colorectal cancer 78, pancreatic cancer 70, cervical carcinoma 79, and ovarian carcinoma 80. Through a meta-analysis, Bovenzi et al. 81 found that higher levels of MCT4 in pan-cancers were associated with poorer clinical prognosis. Therefore, these three key MVIRGs (DBF4, ARG2, and SLC16A3) are closely associated with the tumor and its prognosis, which also proves the correctness of choosing these three MVIRGs to establish a prognostic model to a certain extent.

After modeling these three MVIRGs, we carried out relevant verification. We calculated the risk score of each patient with HCC, and used the median risk score as a cut-off value to classify them into high-risk group or low-risk group. Survival analysis showed that the survival rate of the high-risk group was significantly lower than that of the low-risk group, and the ROC curve also showed that the model performed well in predicting the survival rate of patients with HCC. The correlation analysis between the expression of the three MVIRGs and clinical parameters also showed that the high expression of DBF4 and SLC16A3 genes were significantly related to the low OS of patients with HCC, and the high expression of ARG2 may indicate to a certain extent that the prognosis of patients with HCC is poor. We also conducted a subgroup analysis of the clinical characteristics of the high- and low-risk groups, which showed that the model had significant predictive power for the prognosis of patients with HCC with different clinical characteristics. Multivariate Cox regression analysis showed that the risk score calculated by the model was an independent prognostic indicator, and the ROC curve analysis showed that the risk score had a better predictive value than other conventional clinical parameters. Thus, the excellent predictive value of the model was confirmed again. In addition, we also found consistent trends through survival analysis and ROC curve analysis in the independent data set of the ICGC database, which further verified the reliability and predictive value of the prognostic model. More importantly, we used IHC to perform staining analysis on paraffin sections and found that all three genes in HCC samples had higher expression levels than normal controls. Using qRT-PCR technology and follow-up studies, it was found that the expression of three genes (DBF4, ARG2, and SLC16A3) in HCC samples was increased compared to paired adjacent tissues, and the RFS and OS of those with high expression levels were lower than those with low expression levels. Moreover, we found that the expression levels of these three MVIRGs in MVI-positive HCC samples were higher than that in MVI-negative HCC samples. We standardized the risk score formula of the model and calculated the risk score, which showed that the high risk score was significantly related to the low RFS and OS of patients with HCC. The forest plot analysis found that the high expression of these three MVIRGs and the higher risk scores were more likely to lead to the occurrence of MVI, where the risk score had the greatest impact. These results confirmed that the model not only has good value in terms of survival, but also has important prompting significance for predicting the early recurrence of HCC. This further affirms the clinical utility of this prognostic model at the organizational level. Finally, we classified the conventional clinical parameters into the prognostic model and constructed a nomogram model to achieve the purpose of further specific prediction of individual prognosis.

In summary, we constructed and verified a prognostic model for patients with HCC based on MVI-related genes, and the risk score generated by this model can be used as an independent prognostic indicator and can distinguish patients with different survival outcomes, which has excellent reliability and accuracy. Inevitably, our research had some limitations. First, the results of the research were mainly based on the TCGA and ICGC datasets. Although it has been verified in clinical samples, the sample size needs to be expanded. In contrast, some patients have undergone immune or targeted therapy, which had an impact on the prognosis analysis. Additionally, the potential molecular mechanisms of the three genes we used for modeling lacked further functional experiments *in vivo* or* in vitro*.

## Supplementary Material

Supplementary file 1.

Supplementary file 2.

Supplementary file 3.

Supplementary file 4.

Supplementary file 5.

Supplementary file 6.

## Figures and Tables

**Table 1:** Seven MVIRGs were selected after LASSO regression

Lasso Genes	Coefficient
TPX2	0.06571544
SPA17	0.14942750
TMEM237	0.05985658
DBF4	0.18662863
ZNF836	0.01705960
ARG2	0.15234324
SLC16A3	0.12122064

**Table 2:** Three key MVIRGs (DBF4, ARG2 and SLC16A3) were selected after Stepwise Cox regression analysis

MVIRGs	Coefficient
DBF4	0.400506
ARG2	0.188240
SLC16A3	0.204192

## Abbreviations

HCC: hepatocellular carcinoma
MVI: microvascular invasion
DEGs: difference genes
MVIRGs: MVI-related genes
LIHC: liver hepatocellular carcinoma
DBF4: DBF4 zinc finger
SLC16A3: solute carrier family 16 member 3
ARG2: arginase 2
IHC: immunohistochemistry
qRT-PCR: quantitative real-time polymerase chain reaction
TCGA: the cancer genome atlas
KEGG: kyoto encyclopedia of genes and genomes
ICGC: international cancer genome consortium
GO: gene ontology
LASSO: least absolute shrinkage and selection operator
ROC: receiver operating characteristic
AIC: akaike information criterion
FPKM: fragment per kilobase million
TPM: transcripts per million
BP: biological processes
OS: overall survival
RFS: recurrence-free survival
CI: confidence interval
EMT: epithelial-mesenchymal transition

## References

[1] Sung H, Ferlay J, Siegel RL, Laversanne M, Soerjomataram I, Jemal A (2021). Global Cancer Statistics 2020: GLOBOCAN Estimates of Incidence and Mortality Worldwide for 36 Cancers in 185 Countries. CA: a cancer journal for clinicians.

[2] Heimbach JK, Kulik LM, Finn RS, Sirlin CB, Abecassis MM, Roberts LR (2018). AASLD guidelines for the treatment of hepatocellular carcinoma. Hepatology (Baltimore, Md).

[3] McGlynn KA, Petrick JL, London WT (2015). Global epidemiology of hepatocellular carcinoma: an emphasis on demographic and regional variability. Clinics in liver disease.

[4] Bertuccio P, Turati F, Carioli G, Rodriguez T, La Vecchia C, Malvezzi M (2017). Global trends and predictions in hepatocellular carcinoma mortality. Journal of hepatology.

[5] Liao SH, Chen CL, Hsu CY, Chien KL, Kao JH, Chen PJ (2021). Long-term effectiveness of population-wide multifaceted interventions for hepatocellular carcinoma in Taiwan. Journal of hepatology.

[6] Sun X, Cao Z, Mao K, Wu C, Chen H, Wang J (2020). Photodynamic therapy produces enhanced efficacy of antitumor immunotherapy by simultaneously inducing intratumoral release of sorafenib. Biomaterials.

[7] Santopaolo F, Lenci I, Milana M, Manzia TM, Baiocchi L (2019). Liver transplantation for hepatocellular carcinoma: Where do we stand?. World journal of gastroenterology.

[8] Liang L, Li C, Diao YK, Jia HD, Xing H, Pawlik TM (2020). Survival benefits from adjuvant transcatheter arterial chemoembolization in patients undergoing liver resection for hepatocellular carcinoma: a systematic review and meta-analysis. Therapeutic advances in gastroenterology.

[9] Clavien PA, Lesurtel M, Bossuyt PM, Gores GJ, Langer B, Perrier A (2012). Recommendations for liver transplantation for hepatocellular carcinoma: an international consensus conference report. The Lancet Oncology.

[10] Huang Z, Zhou JK, Peng Y, He W, Huang C (2020). The role of long noncoding RNAs in hepatocellular carcinoma. Molecular cancer.

[11] Forner A, Reig M, Bruix J (2018). Hepatocellular carcinoma. Lancet (London, England).

[12] Fitzmaurice C, Abate D, Abbasi N, Abbastabar H, Abd-Allah F, Abdel-Rahman O (2019). Global, Regional, and National Cancer Incidence, Mortality, Years of Life Lost, Years Lived With Disability, and Disability-Adjusted Life-Years for 29 Cancer Groups, 1990 to 2017: A Systematic Analysis for the Global Burden of Disease Study. JAMA oncology.

[13] Roayaie S, Blume IN, Thung SN, Guido M, Fiel MI, Hiotis S (2009). A system of classifying microvascular invasion to predict outcome after resection in patients with hepatocellular carcinoma. Gastroenterology.

[14] Erstad DJ, Tanabe KK (2019). Prognostic and Therapeutic Implications of Microvascular Invasion in Hepatocellular Carcinoma. Annals of surgical oncology.

[15] Mazzaferro V, Llovet JM, Miceli R, Bhoori S, Schiavo M, Mariani L (2009). Predicting survival after liver transplantation in patients with hepatocellular carcinoma beyond the Milan criteria: a retrospective, exploratory analysis. The Lancet Oncology.

[16] Shen A, Liu M, Zheng D, Chen Q, Wu Z (2020). Adjuvant transarterial chemoembolization after curative hepatectomy for hepatocellular carcinoma with microvascular invasion: A systematic review and meta-analysis. Clinics and research in hepatology and gastroenterology.

[17] Cescon M, Ravaioli M, Grazi GL, Ercolani G, Cucchetti A, Bertuzzo V (2010). Prognostic factors for tumor recurrence after a 12-year, single-center experience of liver transplantations in patients with hepatocellular carcinoma. Journal of transplantation. 2010.

[18] Lee S, Kang TW, Song KD, Lee MW, Rhim H, Lim HK (2021). Effect of Microvascular Invasion Risk on Early Recurrence of Hepatocellular Carcinoma After Surgery and Radiofrequency Ablation. Annals of surgery.

[19] Banerjee S, Wang DS, Kim HJ, Sirlin CB, Chan MG, Korn RL (2015). A computed tomography radiogenomic biomarker predicts microvascular invasion and clinical outcomes in hepatocellular carcinoma. Hepatology (Baltimore, Md).

[20] Wu D, Tan M, Zhou M, Sun H, Ji Y, Chen L (2015). Liver computed tomographic perfusion in the assessment of microvascular invasion in patients with small hepatocellular carcinoma. Investigative radiology.

[21] Du B, Wang F, Jarad B, Wang Z, Zhang Y (2020). A novel signature based on microvascular invasion predicts the recurrence of HCC. Journal of translational medicine.

[22] Wang WT, Yang L, Yang ZX, Hu XX, Ding Y, Yan X (2018). Assessment of Microvascular Invasion of Hepatocellular Carcinoma with Diffusion Kurtosis Imaging. Radiology.

[23] Yang JD, Heimbach JK (2020). New advances in the diagnosis and management of hepatocellular carcinoma. BMJ (Clinical research ed).

[24] Lee SH, Hyun SK, Kim HB, Kang CD, Kim SH (2016). Potential Role of CD133 Expression in the Susceptibility of Human Liver Cancer Stem-Like Cells to TRAIL. Oncology research.

[25] Dimitroulis D, Damaskos C, Valsami S, Davakis S, Garmpis N, Spartalis E (2017). From diagnosis to treatment of hepatocellular carcinoma: An epidemic problem for both developed and developing world. World journal of gastroenterology.

[26] Fitzmaurice C, Allen C, Barber RM, Barregard L, Bhutta ZA, Brenner H (2017). Global, Regional, and National Cancer Incidence, Mortality, Years of Life Lost, Years Lived With Disability, and Disability-Adjusted Life-years for 32 Cancer Groups, 1990 to 2015: A Systematic Analysis for the Global Burden of Disease Study. JAMA oncology.

[27] Jiang YQ, Cao SE, Cao S, Chen JN, Wang GY, Shi WQ (2021). Preoperative identification of microvascular invasion in hepatocellular carcinoma by XGBoost and deep learning. Journal of cancer research and clinical oncology.

[28] Chen SL, Xiao H, Xie ZL, Shen JX, Chen ZB, Wang YQ (2020). The presence of microvascular invasion guides treatment strategy in recurrent HBV-related HCC. European radiology.

[29] Xu X, Zhang HL, Liu QP, Sun SW, Zhang J, Zhu FP (2019). Radiomic analysis of contrast-enhanced CT predicts microvascular invasion and outcome in hepatocellular carcinoma. Journal of hepatology.

[30] Hyun SH, Eo JS, Song BI, Lee JW, Na SJ, Hong IK (2018). Preoperative prediction of microvascular invasion of hepatocellular carcinoma using (18)F-FDG PET/CT: a multicenter retrospective cohort study. European journal of nuclear medicine and molecular imaging.

[31] Lee S, Kim SH, Lee JE, Sinn DH, Park CK (2017). Preoperative gadoxetic acid-enhanced MRI for predicting microvascular invasion in patients with single hepatocellular carcinoma. Journal of hepatology.

[32] Li Y, Zhang Y, Fang Q, Zhang X, Hou P, Wu H (2021). Radiomics analysis of [(18)F]FDG PET/CT for microvascular invasion and prognosis prediction in very-early- and early-stage hepatocellular carcinoma. European journal of nuclear medicine and molecular imaging.

[33] Zhang X, Ruan S, Xiao W, Shao J, Tian W, Liu W (2020). Contrast-enhanced CT radiomics for preoperative evaluation of microvascular invasion in hepatocellular carcinoma: A two-center study. Clinical and translational medicine.

[34] Lim KC, Chow PK, Allen JC, Chia GS, Lim M, Cheow PC (2011). Microvascular invasion is a better predictor of tumor recurrence and overall survival following surgical resection for hepatocellular carcinoma compared to the Milan criteria. Annals of surgery.

[35] Wan T, Zhang T, Si X, Zhou Y (2017). Overexpression of EMT-inducing transcription factors as a potential poor prognostic factor for hepatocellular carcinoma in Asian populations: A meta-analysis. Oncotarget.

[36] Mima K, Hayashi H, Kuroki H, Nakagawa S, Okabe H, Chikamoto A (2013). Epithelial-mesenchymal transition expression profiles as a prognostic factor for disease-free survival in hepatocellular carcinoma: Clinical significance of transforming growth factor-β signaling. Oncology letters.

[37] Zhou YM, Cao L, Li B, Zhang RX, Sui CJ, Yin ZF (2012). Clinicopathological significance of ZEB1 protein in patients with hepatocellular carcinoma. Annals of surgical oncology.

[38] Jin H, He Y, Zhao P, Hu Y, Tao J, Chen J (2019). Targeting lipid metabolism to overcome EMT-associated drug resistance via integrin β3/FAK pathway and tumor-associated macrophage repolarization using legumain-activatable delivery. Theranostics.

[39] Thomas LW, Esposito C, Stephen JM, Costa ASH, Frezza C, Blacker TS (2019). CHCHD4 regulates tumour proliferation and EMT-related phenotypes, through respiratory chain-mediated metabolism. Cancer & metabolism.

[40] Cha YH, Yook JI, Kim HS, Kim NH (2015). Catabolic metabolism during cancer EMT. Archives of pharmacal research.

[41] Schieber MS, Chandel NS (2013). ROS links glucose metabolism to breast cancer stem cell and EMT phenotype. Cancer cell.

[42] Comprehensive and Integrative Genomic Characterization of Hepatocellular Carcinoma Cell. 2017; 169: 1327-41.e23.

[43] Matthews LA, Guarné A (2013). Dbf4: the whole is greater than the sum of its parts. Cell cycle (Georgetown, Tex).

[44] Yeeles JT, Deegan TD, Janska A, Early A, Diffley JF (2015). Regulated eukaryotic DNA replication origin firing with purified proteins. Nature.

[45] Ogi H, Wang CZ, Nakai W, Kawasaki Y, Masumoto H (2008). The role of the Saccharomyces cerevisiae Cdc7-Dbf4 complex in the replication checkpoint. Gene.

[46] Marston AL (2009). Meiosis: DDK is not just for replication. Current biology: CB.

[47] Miller CT, Gabrielse C, Chen YC, Weinreich M (2009). Cdc7p-Dbf4p regulates mitotic exit by inhibiting Polo kinase. PLoS genetics.

[48] Pessoa-Brandão L, Sclafani RA (2004). CDC7/DBF4 functions in the translesion synthesis branch of the RAD6 epistasis group in Saccharomyces cerevisiae. Genetics.

[49] Takayama Y, Mamnun YM, Trickey M, Dhut S, Masuda F, Yamano H (2010). Hsk1- and SCF(Pof3)-dependent proteolysis of S. pombe Ams2 ensures histone homeostasis and centromere function. Developmental cell.

[50] Nambiar S, Mirmohammadsadegh A, Hassan M, Mota R, Marini A, Alaoui A (2007). Identification and functional characterization of ASK/Dbf4, a novel cell survival gene in cutaneous melanoma with prognostic relevance. Carcinogenesis.

[51] Charych DH, Coyne M, Yabannavar A, Narberes J, Chow S, Wallroth M (2008). Inhibition of Cdc7/Dbf4 kinase activity affects specific phosphorylation sites on MCM2 in cancer cells. Journal of cellular biochemistry.

[52] Bonte D, Lindvall C, Liu H, Dykema K, Furge K, Weinreich M (2008). Cdc7-Dbf4 kinase overexpression in multiple cancers and tumor cell lines is correlated with p53 inactivation. Neoplasia (New York, NY).

[53] Cheng AN, Fan CC, Lo YK, Kuo CL, Wang HC, Lien IH (2017). Cdc7-Dbf4-mediated phosphorylation of HSP90-S164 stabilizes HSP90-HCLK2-MRN complex to enhance ATR/ATM signaling that overcomes replication stress in cancer. Scientific reports.

[54] Dimitriades V, Rodriguez PC, Zabaleta J, Ochoa AC (2014). Arginase I levels are decreased in the plasma of pediatric patients with atopic dermatitis. Annals of allergy, asthma & immunology: official publication of the American College of Allergy, Asthma, & Immunology.

[55] Vonk JM, Postma DS, Maarsingh H, Bruinenberg M, Koppelman GH, Meurs H (2010). Arginase 1 and arginase 2 variations associate with asthma, asthma severity and beta2 agonist and steroid response. Pharmacogenetics and genomics.

[56] Grzywa TM, Sosnowska A, Matryba P, Rydzynska Z, Jasinski M, Nowis D (2020). Myeloid Cell-Derived Arginase in Cancer Immune Response. Frontiers in immunology.

[57] Ino Y, Yamazaki-Itoh R, Oguro S, Shimada K, Kosuge T, Zavada J (2013). Arginase II expressed in cancer-associated fibroblasts indicates tissue hypoxia and predicts poor outcome in patients with pancreatic cancer. PloS one.

[58] Sousa MS, Latini FR, Monteiro HP, Cerutti JM (2010). Arginase 2 and nitric oxide synthase: Pathways associated with the pathogenesis of thyroid tumors. Free radical biology & medicine.

[59] Zhang J, Jin Y, Xu S, Zheng J, Zhang QI, Wang Y (2016). AGR2 is associated with gastric cancer progression and poor survival. Oncology letters.

[60] Mussai F, Egan S, Hunter S, Webber H, Fisher J, Wheat R (2015). Neuroblastoma Arginase Activity Creates an Immunosuppressive Microenvironment That Impairs Autologous and Engineered Immunity. Cancer research.

[61] Fultang L, Gamble LD, Gneo L, Berry AM, Egan SA, De Bie F (2019). Macrophage-Derived IL1β and TNFα Regulate Arginine Metabolism in Neuroblastoma. Cancer research.

[62] Bron L, Jandus C, Andrejevic-Blant S, Speiser DE, Monnier P, Romero P (2013). Prognostic value of arginase-II expression and regulatory T-cell infiltration in head and neck squamous cell carcinoma. International journal of cancer.

[63] Mussai F, De Santo C, Abu-Dayyeh I, Booth S, Quek L, McEwen-Smith RM (2013). Acute myeloid leukemia creates an arginase-dependent immunosuppressive microenvironment. Blood.

[64] Zaytouni T, Tsai PY, Hitchcock DS, DuBois CD, Freinkman E, Lin L (2017). Critical role for arginase 2 in obesity-associated pancreatic cancer. Nature communications.

[65] Martí i Líndez AA, Dunand-Sauthier I, Conti M, Gobet F, Núñez N, Hannich JT (2019). Mitochondrial arginase-2 is a cell-autonomous regulator of CD8+ T cell function and antitumor efficacy. JCI insight.

[66] Ochocki JD, Khare S, Hess M, Ackerman D, Qiu B, Daisak JI (2018). Arginase 2 Suppresses Renal Carcinoma Progression via Biosynthetic Cofactor Pyridoxal Phosphate Depletion and Increased Polyamine Toxicity. Cell metabolism.

[67] Halestrap AP (2013). The SLC16 gene family - structure, role and regulation in health and disease. Molecular aspects of medicine.

[68] Fisel P, Kruck S, Winter S, Bedke J, Hennenlotter J, Nies AT (2013). DNA methylation of the SLC16A3 promoter regulates expression of the human lactate transporter MCT4 in renal cancer with consequences for clinical outcome. Clinical cancer research: an official journal of the American Association for Cancer Research.

[69] Choi SH, Kim MY, Yoon YS, Koh DI, Kim MK, Cho SY (2019). Hypoxia-induced RelA/p65 derepresses SLC16A3 (MCT4) by downregulating ZBTB7A. Biochimica et biophysica acta Gene regulatory mechanisms.

[70] Baek G, Tse YF, Hu Z, Cox D, Buboltz N, McCue P (2014). MCT4 defines a glycolytic subtype of pancreatic cancer with poor prognosis and unique metabolic dependencies. Cell reports.

[71] Baenke F, Dubuis S, Brault C, Weigelt B, Dankworth B, Griffiths B (2015). Functional screening identifies MCT4 as a key regulator of breast cancer cell metabolism and survival. The Journal of pathology.

[72] Zhang L, Zhang Z, Yu Z (2019). Identification of a novel glycolysis-related gene signature for predicting metastasis and survival in patients with lung adenocarcinoma. Journal of translational medicine.

[73] Yu S, Wu Y, Li C, Qu Z, Lou G, Guo X (2020). Comprehensive analysis of the SLC16A gene family in pancreatic cancer via integrated bioinformatics. Scientific reports.

[74] Fan S, Tang J, Li N, Zhao Y, Ai R, Zhang K (2019). Integrative analysis with expanded DNA methylation data reveals common key regulators and pathways in cancers. NPJ genomic medicine.

[75] Nahm JH, Kim HM, Koo JS (2017). Glycolysis-related protein expression in thyroid cancer. Tumour biology: the journal of the International Society for Oncodevelopmental Biology and Medicine.

[76] Gerlinger M, Santos CR, Spencer-Dene B, Martinez P, Endesfelder D, Burrell RA (2012). Genome-wide RNA interference analysis of renal carcinoma survival regulators identifies MCT4 as a Warburg effect metabolic target. The Journal of pathology.

[77] Gao HJ, Zhao MC, Zhang YJ, Zhou DS, Xu L, Li GB (2015). Monocarboxylate transporter 4 predicts poor prognosis in hepatocellular carcinoma and is associated with cell proliferation and migration. Journal of cancer research and clinical oncology.

[78] Pinheiro C, Longatto-Filho A, Azevedo-Silva J, Casal M, Schmitt FC, Baltazar F (2012). Role of monocarboxylate transporters in human cancers: state of the art. Journal of bioenergetics and biomembranes.

[79] Pinheiro C, Longatto-Filho A, Ferreira L, Pereira SM, Etlinger D, Moreira MA (2008). Increasing expression of monocarboxylate transporters 1 and 4 along progression to invasive cervical carcinoma. International journal of gynecological pathology: official journal of the International Society of Gynecological Pathologists.

[80] Elsnerova K, Mohelnikova-Duchonova B, Cerovska E, Ehrlichova M, Gut I, Rob L (2016). Gene expression of membrane transporters: Importance for prognosis and progression of ovarian carcinoma. Oncology reports.

[81] Bovenzi CD, Hamilton J, Tassone P, Johnson J, Cognetti DM, Luginbuhl A (2015). Prognostic Indications of Elevated MCT4 and CD147 across Cancer Types: A Meta-Analysis. BioMed research international.

