# Supplementary material for: Identification and Validation of a Prognostic Model Based on Three MVI-Related Genes in Hepatocellular Carcinoma
Source: Int J Biol Sci. 2022 Jan 1;18(1):261–75. doi: 10.7150/ijbs.66536 (PMC8692135; doi:10.7150/ijbs.66536)
Supplement: Supplementary file 1 [file ijbsv18p0261s1.pdf]

DBF4:
Forward Primer: 5’- GGGCAAAAGAGTTGGTAGTGG -3’
Reverse Primer: 5’- ACTTATCGCCATCTGTTTGGATT -3’

ARG2:
Forward Primer: 5’- ACCTGATAGTGAATCCACGCT - 3’
Reverse Primer: 5’- CATGGGCATCAACCCAGAC -3’

SLC16A3:
Forward Primer: 5’- CGGCTTTGTGCTTTACGCC -3’
Reverse Primer: 5’- GCTGAAGAGGTAGACGGAGTA -3’

GAPDH:
Forward Primer: 5’- GGAGCGAGATCCCTCCAAAAT -3’
Reverse Primer: 5’- GGCTGTTGTCATACTTCTCATGG -3’
